# Great Ascitic Effusion

**Published:** 1836

**Authors:** J. B. Beall

**Affiliations:** Missouri


*Notes of a case of Ascites, in which an extraordinary quantity of serum was drawn off by repeated Tappings—Communicated by* Dr. J. B. Beall, of Missouri.

The following tabular view of the number of Tappings, and the quantity of water drawn off by each, was kept by the patient himself in conjunction with his friends. His name was James Peuquite, late of Clinton county, Missouri, now deceased.

TABLE.

| Dates. | Tapping. | Quantity. Gals. | Qts. | 1835. | | Gals. | Qts. |
|---|---|---|---|---|---|---|---|
| To Aug. 18, 1833 | 15 " | 74 | 0 | July, 3 | 1 Tapping | 2 | 0 |
| To Jan. 18, 1835 | 33 " | 92 | 0 | 7 | 1 " | 2 | 0 |
| " " 21 | 1 " | 2 | 0 | 13 | 1 " | 2 | 3 |
| 28 | 1 " | 2 | 2 | 18 | 1 " | 2 | 2 |
| Feb. 4 | 1 " | 2 | 2 | 23 | 1 " | 2 | 1 |
| 6 | Br. and run. | 0 | 3 | 28 | 1 " | 2 | 2 |
| 11 | 1 Tap. | 2 | 1 | August, 3 | 1 " | 2 | 1 |
| 17 | 1 " | 2 | 2 | 9 | 1 " | 2 | 1 |
| 23 | 1 " | 2 | 2 | 14 | 1 " | 2 | 0 |
| 27 | 1 " | 2 | 0 | 19 | 1 " | 1 | 2 |
| March, 5 | 1 " | 2 | 1 | Sept. 10 | 1 " | 2 | 2 |
| 11 | 1 " | 2 | 0 | 16 | 1 " | 3 | 0 |
| 17 | 1 " | 2 | 2 | 25 | 1 " | 2 | 2 |
| 22 | 1 " | 1 | 2 | Oct. 3 | 1 " | 2 | 0 |
| 29 | 1 " | 2 | 2 | 11 | 1 " | 2 | 2 |
| April, 3 | 1 " | 2 | 0 | 23 | 1 " | 2 | 0 |
| 10 | 1 " | 2 | 2 | Nov. 5 | 1 " | 2 | 1 |
| 16 | 1 " | 2 | 2 | 19 | 1 " | 2 | 1 |
| 21 | 1 " | 2 | 1 | Dec. 4 | 1 " | 2 | 0 |
| 26 | 1 " | 2 | 1 | 13 | 1 " | 2 | 2 |
| May, 1 | 1 " | 2 | 0 | | | | |
| 6 | 1 " | 1 | 3 | | | | |
| 12 | 1 " | 2 | 0 | | | | |
| 17 | 1 " | 2 | 1 | | | | |
| 24 | 1 " | 2 | 3 | | | | |
| 25 | Br. and run | 0 | 3 | | | | |
| 30 | 1 " | 2 | 0 | | | | |
| June, 4 | 1 " | 2 | 0 | | | | |
| 10 | 1 " | 2 | 2 | | | | |
| 16 | 1 " | 2 | 3 | | | | |
| 23 | 1 " | 2 | 1 | | | | |
| 28 | 1 " | 2 | 1 | | 96 | 275 | 2 |

To give a detailed account of the treatment of this case, as it proved fatal, I presume is unnecessary. Previous to my seeing him, he had been under the care of several eminent physicians, one of Kentucky, and one of Cincinnati with others now forgotten. When I was called to him in the 5th month, (May) 1834, he was in a very reduced state; for the greatest part of the time confined to bed. His liver was deranged, spleen enlarged and indurated, chronic diarrhœa, hæmorrhoids, and what added greatly to his suffering he had a large inguinal hernia which could not be kept up by a truss, and hence it demanded his attention every few minutes to reduce it. I first put him on a course of astringents and tonics; after the lapse of a few weeks he found himself relieved from diarrhœa and hæmorrhoids. I now paid attention to the disease of the liver and spleen. Mercury, iodine, etc., with diruetics were amply tried without producing any evident beneficial effect. The first fifteen tappings produced an average quantity of water of a fraction less than five gallons to each operation. Subsequently the tappings were performed in a rupture of the umbilicus which gave no pain, the skin being the only part to puncture, which at any time he could do himself, consequently the tappings were more frequently performed than they had been before, and less obtained at a time. The largest quantity was three gallons. From the 1st of 8th month, (August) the water did not collect so fast in the abdomen as it had before. He then felt better than he at any time previous, since he had been affected, was able to drive a waggon, and to do many light jobs of work on his farm. About two weeks before his decease, he came to see me, a distance of 10 miles. The spleen was then more reduced than it had ever been, his health improved, and he flattered himself he would recover, but soon after he returned home he was taken with sick stomach and vomiting, (occasioned perhaps by too long exposure to cold damp air and over fatigue,) which continued daily until the 13th December, at which time he was greatly reduced in flesh and strength. Under these unfavorable circumstances he was imprudently tapped, and it brought on syncope, from which he never recovered.

I did not see him when he had sickness and vomiting. For relief, he depended altogether on his own judgment in the use of such means as he had, or procured himself. On the day he was tapped the last time, I was called. On my arrival at his house, perhaps eight or ten hours after the operation was performed, I found him dying. He expired in a few hours.

